# Lanthanum and Manganese Co-Doping Effects on Structural, Morphological, and Magnetic Properties of Sol-Gel Derived BiFeO_3_

**DOI:** 10.3390/ma14174844

**Published:** 2021-08-26

**Authors:** Dovydas Karoblis, Ramunas Diliautas, Kestutis Mazeika, Dalis Baltrunas, Gediminas Niaura, Martynas Talaikis, Aldona Beganskiene, Aleksej Zarkov, Aivaras Kareiva

**Affiliations:** 1 Institute of Chemistry, Vilnius University, Naugarduko 24, LT-03225 Vilnius, Lithuania; dovydas.karoblis@chgf.vu.lt (D.K.); ramunas.diliautas@chgf.stud.vu.lt (R.D.); aldona.beganskiene@chgf.vu.lt (A.B.); aleksej.zarkov@chgf.vu.lt (A.Z.); 2 Center of Physical Sciences and Technology, LT-02300 Vilnius, Lithuania; kestas@ar.fi.lt (K.M.); dalis@ar.fi.lt (D.B.); gediminas.niaura@ftmc.lt (G.N.); 3 Institute of Chemical Physics, Faculty of Physics, Vilnius University, Sauletekio Ave. 3,LT-10257 Vilnius, Lithuania; 4 Department of Bioelectrochemistry and Biospectroscopy, Institute of Biochemistry, Life Sciences Center, Vilnius University, LT-10257 Vilnius, Lithuania; martynas.talaikis@gmc.vu.lt

**Keywords:** bismuth ferrite, lanthanum ferrite, solid solution, sol-gel processing, antiferromagnetism

## Abstract

In this work, lanthanum and manganese co-substitution effects on different properties of bismuth ferrite solid solutions Bi_1-x_La_x_Fe_0.85_Mn_0.15_O_3_ (x from 0 to 1) prepared by a sol-gel synthetic approach have been investigated. It was observed that the structural, morphological, and magnetic properties of obtained specimens are influenced by the amount of introduced La^3+^ ions. Surprisingly, only the compound with a composition of BiFe_0.85_Mn_0.15_O_3_ was not monophasic, and the presence of neighboring phases was determined from X-ray diffraction analysis and Mössbauer measurements. Structural transitions from orthorhombic to cubic and back to orthorhombic were also observed depending on the La^3+^ amount. Antiferromagnetic behaviour was observed for all of the samples, with the highest magnetisation values for Bi_0.5_La_0.5_Fe_0.85_Mn_0.15_O_3_. Additionally, structural attributes and morphological features were evaluated by Raman spectroscopy and scanning electron microscopy (SEM), respectively.

## 1. Introduction

Since the work of Spaldin in 2000, where the existence of only a few magnetic ferroelectrics was questioned [1], multiferroics became one of the most investigated topics in the past 20 years. In this type of material, two of the three primary ferroic (ferroelectricity, ferromagnetism, or ferroelasticity) properties coincide in one phase. The combination of magnetism and ferroelectricity are interesting from both theoretical and application point of views. From the practical side, these compounds could be applied in energy-efficient devices [2], photocatalysis [3], biomedicine [4], microwave phase shifter [5], etc. From the theoretical aspect, magnetism arises from partially filled d or f shells, while ferroelectricity is associated with the off-centring of different transition metal ions, such as Ti^4+^, where the d orbital is empty. Therefore, few mechanisms (lone pair, charge ordering, or “geometric”) explaining ferroelectricity are known [6].

Bismuth ferrite (BiFeO_3_) can be considered one of the most investigated perovskite-type multiferroic materials. This compound demonstrates magnetoelectric properties in both thin-film [7] and bulk [8] forms. Nanosized BiFeO_3_ structures, which display different morphologies, are also explored thoroughly since these nanostructures showed an increase in magnetisation values [9] and better photocatalytic activity [10]. The combination of both ferroelectric and (anti)ferromagnetic orders remains a difficult challenge for this compound due to the large leakage current, which arises from high oxygen vacancies concentration, weak magnetoelectric effect, and formation of impurities [11]. Another material, which can display both spontaneous polarisation and magnetisation with magnetoelectric effect, is lanthanum ferrite (LaFeO_3_) [12]. While phase-pure BiFeO_3_ is difficult to prepare, LaFeO_3_ can be synthesised by different types of techniques, including sol-gel combustion [13], solid-state [14], hydrothermal [15], co-precipitation, or high-energy ball milling [16].

Substitution of A-site, B-site, or both cations can lead to improvement in physical properties or formation of phase-pure BiFeO_3_. One of the examples can be La^3+^ intercalation to the BiFeO_3_ system, leading to an increase in magnetisation, conductivity, and improved ferroelectric properties [17,18,19]. Substituting Fe^3+^ ions with Mn^3+^ ions can lead to structural transition, a better magnetoelectric coupling effect, and enhancement of magnetisation [20,21,22]. Co-doping with both elements was performed to suppress the formation of oxygen vacancies and Fe^2+^ ions, which could improve the electrical properties [23]. Furthermore, it was shown that Bi_1−x_La_x_Fe_1−y_Mn_y_O_3_/Ti_3_C_2_ hybrids have high catalytic activity towards Congo Red degradation [24]. While most of the studies regarding Bi_1-x_La_x_Fe_1-y_Mn_y_O_3_ focus on intercalation of small amounts of La^3+^ up to 30% [25], we aimed to investigate the whole compositional range.

In this work, we prepared a series of Bi_1-x_La_x_Fe_0.85_Mn_0.15_O_3_ (with different x steps) solid solutions by an environmentally friendly, cost-effective, and simple sol-gel technique using only ethylene glycol as a complexing agent. Various characterisation techniques were used to evaluate structural, morphological, and magnetic properties regarding chemical composition.

## 2. Materials and Methods

For the preparation of Bi_1-x_La_x_Fe_0.85_Mn_0.15_O_3_ solid solutions, bismuth (III) nitrate pentahydrate (Bi(NO_3_)_3_∙5H_2_O, Roth (Karlsruhe, Germany), 98%), lanthanum (III) nitrate hexahydrate (La(NO_3_)_3_∙6H_2_O, Alfa Aesar (Haverhill, MA, USA), 99.9%), iron (III) nitrate nonahydrate (Fe(NO_3_)_3_∙9H_2_O, Alfa Aesar, 99.9%), and manganese (II) nitrate tetrahydrate (Mn(NO_3_)_2_∙4H_2_O, Alfa Aesar, 99.9%) were used as starting materials. Firstly, required amounts of lanthanum, iron, and manganese nitrates were dissolved in 20 mL of distilled water. Before addition of bismuth nitrate, the pH of solution was adjusted to 1 by the addition of nitric acid (HNO_3_, 65%). After the dissolution of bismuth nitrate, the complexing agent, ethylene glycol (C_2_H_6_O_2_, Sigma-Aldrich (St. Louis, MO, USA), ≥99.5%), was added to the mixture. The molar ratio between total metal ions and ethylene glycol was 1:2. The obtained solution was homogenised under constant stirring at 90 °C for 1 h. Next, the temperature was increased to 120 °C for solvent evaporation and the formation of a gel. The resulting gel was dried in the oven at 120 °C for 6 h, ground in agate mortar, and annealed at 650 °C with a heating rate of 1 °C/min for 1.5 h in air.

Thermal decomposition of precursor gel was investigated by thermogravimetric and differential scanning calorimetric (TG/DTG-DSC) analysis using PerkinElmer STA 6000 Simultaneous Thermal Analyzer. About 5–10 mg of dried sample was heated from 30 °C to 900 °C at 10 °C/min heating rate in dry flowing air (20 mL/min). X-ray diffraction (XRD) analysis of obtained products was performed with a Rigaku Miniflex II diffractometer using a primary beam Cu Kα radiation (λ = 1.541838 Å). The 2θ angle of the diffractometer was set in the range from 20° to 70° while moving 10°/min. The obtained diffraction data were refined by the Rietveld method using the Fullprof suite. Crystallite size was calculated by applying Scherrer’s equation: $D=\frac{K\lambda }{\beta cos\theta }$, with the following: K–shape factor (in our case, 0.89), *λ*–X-ray wavelength, *β*–full width at half maximum in radian, θ–Bragg diffraction angle. The β was measured for corundum standard in order to evaluate the instrumental broadening. Alpha FT-IR spectrometer (Bruker, Ettlingen, Germany) was used for FT-IR analysis of compounds. All spectra were recorded at ambient temperature in the range of 4000–400 cm^−1^. The morphology of samples was examined using a scanning electron microscope (SEM) (Hitachi SU-70, Tokyo, Japan). Raman spectra were acquired using LabRam HR800 (Horiba Jobin Yvon, Villeneuve d′Ascq, France) Raman spectrometer equipped with thermoelectrically cooled (−90 °C) CCD camera (DU920P-BR-DD), 600 lines/mm grating and microscope. Spectra were excited with a 532 nm beam from the CW diode-pumped solid-state (DPSS) laser (Cobolt Samba, Hübner Photonics, Stockholm, Sweden). The laser power at the sample was restricted to 0.2 mW to avoid laser-induced sample heating and photodegradation. The 50×/0.50 NA long working distance (LWD) objective was employed during the measurements. The overall integration time was 600 s. The position of the Raman bands on the wavenumber axis was calibrated by the Si Raman band at 520.7 cm^−1^. Parameters of the bands were determined by fitting the experimental spectra with Gaussian-Lorentzian shape components using GRAMS/A1 8.0 (Thermo Scientific, Waltham, MA, USA) software. Magnetometer consisting of the lock-in amplifier SR510 (Stanford Research Systems, Sunnyvale, CA, USA), the Gauss/teslameter FH-54 (Magnet Physics) and the laboratory magnet supplied by the power source SM 330-AR-22 (Delta Elektronika, Zierikzee, The Netherlands) was applied to record magnetisation dependences on an applied magnetic field. Mössbauer spectra were measured using ^57^Co(Rh) source and Mössbauer spectrometer (Wissenschaftliche Elektronik GmbH, Starnberg, Germany). Closed-cycle He cryostat (Advanced Research Systems, Macungie, PA, USA) was applied for low-temperature measurements. The doublets/singlets, sextets, and hyperfine field distributions were used to fit Mössbauer spectra applying WinNormos Site and Dist software. Isomer shift is given relative to α-Fe.

## 3. Results

**Figure 1:**
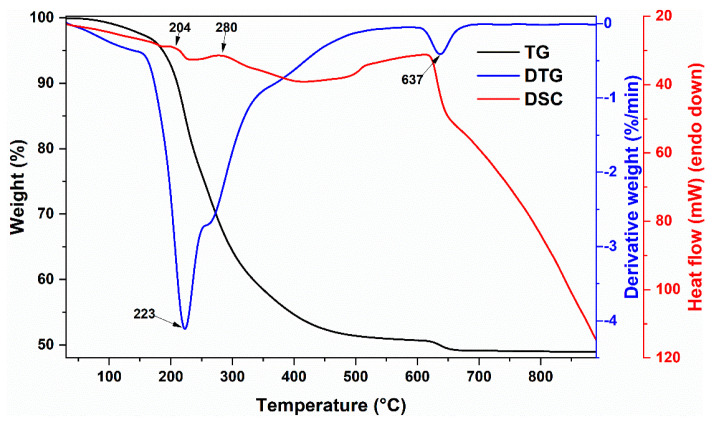
TG/DTG/DSC curves of (0.1)Bi-(0.9)La-(0.85)Fe-(0.15)Mn gel.

Thermogravimetric analysis was employed to evaluate thermal decomposition behaviour and determine optimum annealing temperature, which is required for the formation of solid solutions. TG/DTG/DSC curves of precursor gel for Bi_0.1_La_0.9_Fe_0.85_Mn_0.15_O_3_ composition compound are presented in Figure 1. Three main degradation steps can be identified from the DTG curve. First, non-significant mass loss (about 3%) can be observed in the 60–150 °C temperature range; it can be ascribed to evaporation of adsorbed water. The most significant mass loss (about 46%) can be seen in the 160–500 °C temperature region. This step can be attributed to several processes, including the decomposition of metal nitrates or metal complexes with ethylene glycol and the residual organic part of the gel. Two small exothermic peaks, which are centred around 204 and 280 °C, are the result of a combustion reaction. Final mass loss (about 2%) can be seen in the 625–650 °C range. A similar degradation step previously was observed for La–Fe–O gel and was explained by the decomposition of residual organic compounds [26], ionic carbonates [27], or degradation of amorphous material [28]. From the TG curve, the total mass loss was determined to be 51%, and above 650 °C, the mass remained constant. For this reason, 650 °C was chosen as a final annealing temperature.

**Figure 2:**
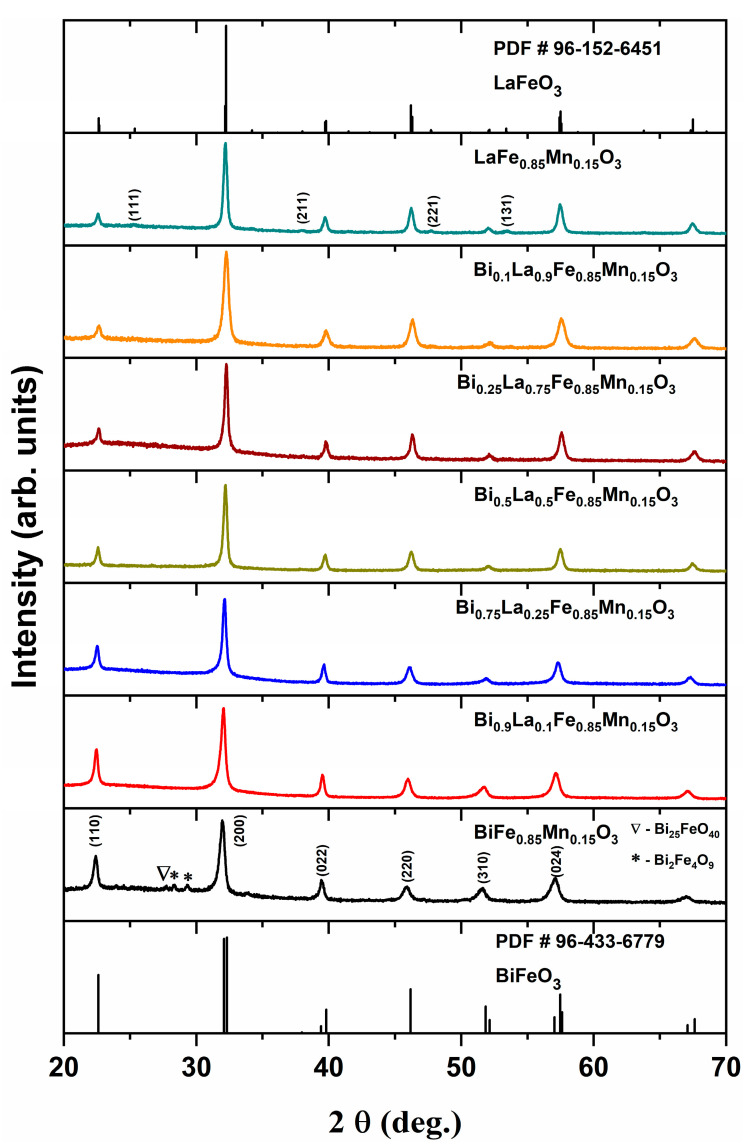
XRD patterns of Bi_1-x_La_x_Fe_0.85_Mn_0.15_O_3_ samples.

X-ray diffraction analysis was carried out to determine phase purity along with structural changes for the Bi_1-x_La_x_Fe_0.85_Mn_0.15_O_3_ solid solutions. The obtained results are demonstrated in Figure 2. Nearly all samples were identified as phase-pure with one exception—pristine BiFe_0.85_Mn_0.15_O_3_. It is known that Fe^3+^ substitution by the small amount of Mn^3+^ can lead to stabilisation of the perovskite phase while avoiding the formation of neighbouring phases [29]. On the other hand, the annealing temperature, which was used in this work, belongs to the range where BiFeO_3_ is considered to be metastable with respect to two Bi-rich Bi_25_FeO_39_ sillenite and Fe-rich Bi_2_Fe_4_O_9_ mullite phases [30]. Those two impurities phases were observed in our synthesised BiFe_0.85_Mn_0.15_O_3_ sample. By intercalating La^3+^ into the perovskite structure, few trends can be observed in the XRD profiles. The intensity of the peaks labelled (110), (022), and (310) decreases, while the intensity of the (220) peak increases for La-rich solid solutions. Since the ionic radius of the La^3+^ ion (ionic radius 1.16 Å in VIII-fold coordination) is slightly smaller than the Bi^3+^ ion (ionic radius 1.17 Å in VIII-fold coordination) [31], only a slight peak shift can be observed in the XRD patterns.

Rietveld refinement was carried out for all synthesised solid solution specimens. The splitting of the most intense (200) peak was not observed for any of our samples, which means that the perovskite structure deviates from the crystal symmetry of the BiFeO_3_ compound, which has a trigonal unit cell with rhombohedral or hexagonal axes. Fitting was performed with various structural models (*R3c, Pbnm, Pm*$\bar{3}$*m, R3c*+*Pbnm, R3c*+*Pm*$\bar{3}$*m*, etc.), previously completed by Kumar and Kar [25] for Bi_1-x_La_x_Fe_1-x_Mn_x_O_3_ (x varying from 0 to 0.3) solid solutions. They observed that the structure of all co-doped samples consists of a mixture of two different phases: orthorhombic with *Pbnm* space group and rhombohedral with *R3c* space group. In our case, the BiFe_0.85_Mn_0.15_O_3_ sample has an orthorhombic unit cell with a *Pbnm* space group. B-site substitution with Mn^3+^ [32] or Ga^3+^ [33] ions can lead to identical structural transition. The compound with 10% La^3+^ has an identical structure to the BiFe_0.85_Mn_0.15_O_3_ sample, with a decrease in cell volume and slight variations in lattice parameters. Further increase in La^3+^ up to 75% resulted in a structural change to a cubic unit cell with *Pm*$\bar{3}$*m* space group. Moreover, the appearance of new diffractions peaks labelled (111), (211), (221), and (131) for a sample containing 90% of La^3+^ confirms another structural transition from cubic to the orthorhombic unit cell with the *Pbnm* space group. Interestingly, in comparison with the solid solution with 90% of La^3+^ ions, the LaFe_0.85_Mn_0.15_O_3_ compound has an increase in unit cell parameters and cell volume. Analogous results were observed for Bi_1-x_La_x_FeO_3_ solid solutions, where an increase in La^3+^ concentration resulted in an increase in cell parameters [34]. This was explained by the fact that Bi-containing compounds have a larger quantity of oxygen vacancies because of the lower strength of the Bi–O bond.

The most intense diffraction peak (200) was chosen for the calculation of crystallite size. The obtained results are presented in Table 1. Three distinctive regions can be excluded according to the structural transitions observed from XRD patterns. Firstly, the intercalation of 10% of La^3+^ leads to a small increase in crystallite size. After the structural change from orthorhombic to cubic unit cell, the crystallite size increased by 25% in comparison with the previous compound. The compound with 90% of La^3+^ showed a sudden decrease in crystallite size, which can be associated with the structural change. Furthermore, LaFe_0.85_Mn_0.15_O_3_ showed a similar crystallite size to the Bi_0.25_La_0.75_Fe_0.85_Mn_0.15_O_3_ solid solution.

**Figure 3:**
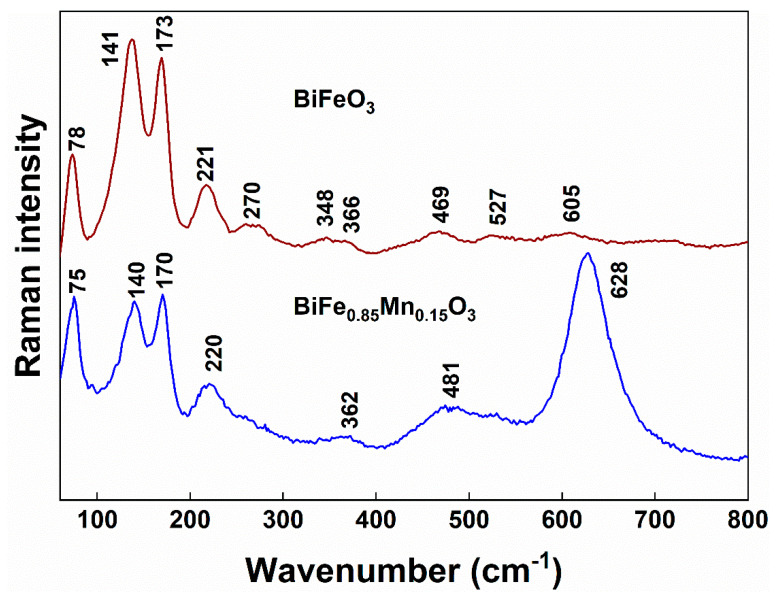
Raman spectra of polycrystalline BiFeO_3_ and BiFe_0.85_Mn_0.15_O_3_ samples in the frequency region of 60−800 cm^−1^. The excitation wavelength is 532 nm (0.2 mW).

The short-range structure of studied compounds was probed by Raman spectroscopy by using a 532 nm excitation wavelength. The technique is very sensitive to structural distortions and could provide additional structural information on unit cells as well as space-group type. Figure 3 compares the Raman spectra of BiFeO_3_ and BiFe_0.85_Mn_0.15_O_3_ samples. BiFe_0.85_Mn_0.15_O_3_ was chosen because this compound is known for possessing different structures with a variety of space groups. Pure BiFeO_3_ exhibits a characteristic pattern of bands related to a rhombohedrically distorted perovskite structure; the main bands are located at 78 cm^−1^ (symmetry E, band E-1), 141 cm^−1^ (A_1_-1), 173 cm^−1^ (A_1_-2), 221 cm^−1^ (A_1_-3), 270 cm^−1^ (E-3), 348 cm^−1^ (E-5), 366 cm^−1^ (E-6), 469 cm^−1^ (A_1_-5), and 527 cm^−1^ (E-8) [29,35,36,37,38]. The low-intensity band near 604 cm^−1^ might be associated with the E-9 mode or related with second-order vibrational transition [38,39,40]. The A_1_ symmetry bands located at 141, 173, and 221 cm^−1^ and one E symmetry mode located at 78 cm^−1^ are the most intense features in the spectrum of pure BiFeO_3_ compound.

Doping with Mn^3+^ ions results in drastic changes in the Raman spectrum (Figure 3). A strong and relatively broad band appears near 628 cm^−1^ along with the broad middle-intensity feature near 481 cm^−1^. In contrast, little changes are visible for lower frequency A_1_ symmetry bands located at 140, 170, and 220 cm^−1^. These low-frequency modes are mainly related to Bi−O stretching vibrations [41,42]. Thus, small spectra changes indicate little perturbation in the Bi−O bonding structure after the introduction of Mn^3+^ ions. The appearance of a strong band near 628 cm^−1^ indicates Mn^3+^ ions-induced perturbations in the Fe−O bonding range. Indeed, high-frequency E-8 and E-9 modes earlier were attributed as mainly related to Fe−O stretching bands [41,42]. These peaks are sensitive to the tilt of oxygen octahedra [40]. Intensification of a band at 628 cm^−1^ might be related to the transformation of short-range structure from rhombohedral (*R3c* space group) to orthorhombic (*Pbnm* space group) [43]. A similar band was observed for orthorhombic rare-earth manganites (RMnO_3_) (*Pnma* space group for oxygen octahedra) [44]. These observations confirm the results of the XRD analysis.

**Figure 4:**
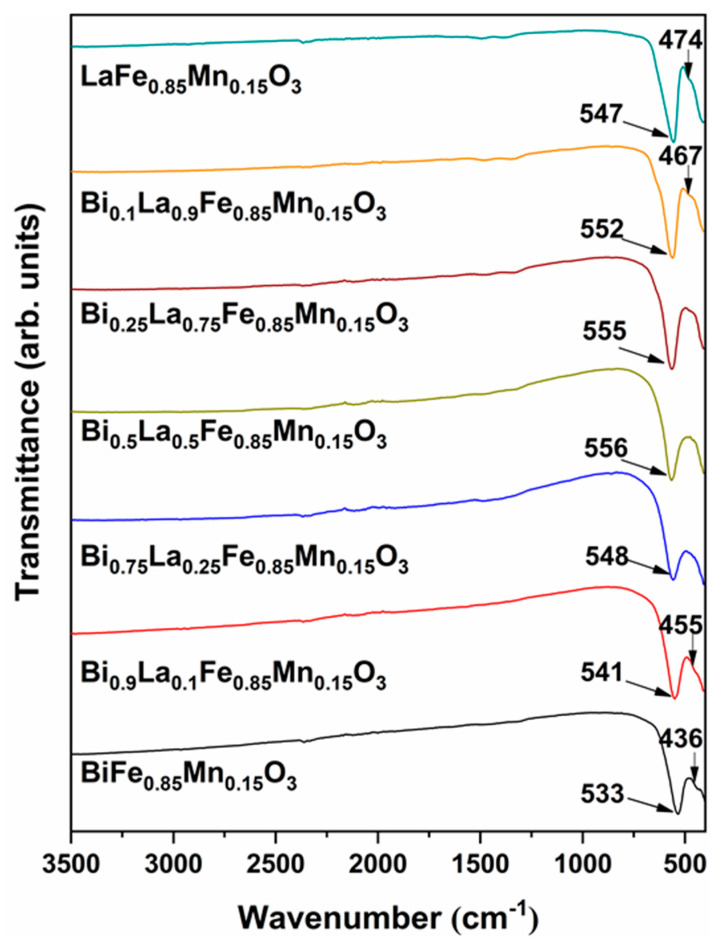
FT-IR spectra of Bi_1-x_La_x_Fe_0.85_Mn_0.15_O_3_ samples.

FT-IR spectroscopy analysis was carried out to further investigate structural changes in Bi_1-x_La_x_Fe_0.85_Mn_0.15_O_3_ solid solutions, and results are demonstrated in Figure 4. In general, the characteristic bands for orthoferrites and orthomanganites can be found in slightly different regions—600–250 cm^−1^ and 700–200 cm^−1^, respectively [45]. In our case, no absorption bands were observed in the 4000–600 cm^−1^ interval, which indicates the absence of residual organic species or carbonates in solid solutions. Our previous study [22] on BiFe_1-x_Mn_x_O_3_ solid solutions revealed that the addition of Mn^3+^ could result in the most intense peak shift to higher wavenumbers. Samples with orthorhombic structure have two absorption bands: a strong and sharp band located at 533–552 cm^−1^ region as well as a weak and broad one at 436–474 cm^−1^. Only one peak at around 550 cm^−1^ can be observed for compounds with cubic structures. These bands can be ascribed to the stretching mode of the Fe–O bond in the FeO_6_ octahedra. With the increase of La^3+^ concentration, weaker absorption band shifts to higher wavenumbers, while a strong intensity peak only shifts to higher wavenumbers until the La^3+^ amount reaches 50%. Larger substitution results in a monotonic shift to lower wavenumbers. Furthermore, no stretching or bending vibrations belonging to Bi_2_O_3_ or La_2_O_3_ can be visible for all our samples, which indicate no presence of these impurity phases.

**Figure 5:**
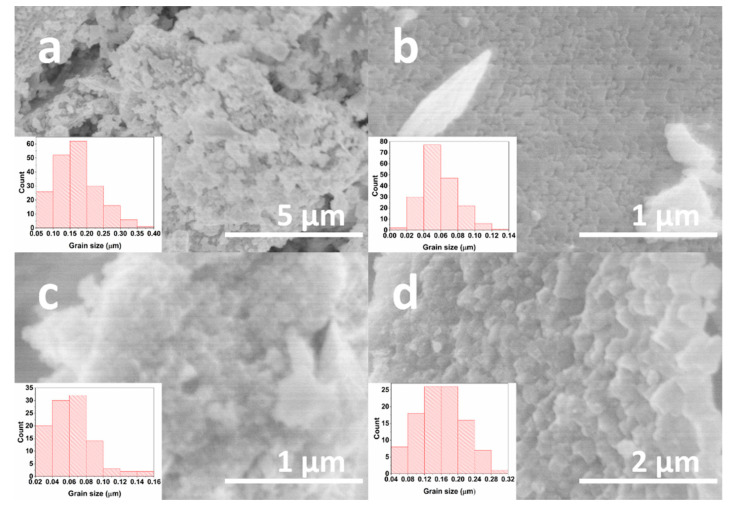
SEM images and grain size distribution of Bi_0.9_La_0.1_Fe_0.85_Mn_0.15_O_3_. (**a**), Bi_0.75_La_0.25_Fe_0.85_Mn_0.15_O_3_ (**b**), Bi_0.5_La_0.5_Fe_0.85_Mn_0.15_O_3_ (**c**) and Bi_0.1_La_0.9_Fe_0.85_Mn_0.15_O_3_ (**d**) solid solutions.

To investigate the influence of La^3+^ and Mn^3+^ co-doping on the morphology of mixed-metal ferrite powders, the SEM analysis was executed for solid solutions of four different compositions, and the results are demonstrated in Figure 5. The Bi_0.9_La_0.1_Fe_0.85_Mn_0.15_O_3_ sample (Figure 5a) consists of particles, which are necked to each other, forming some larger aggregates. Around 90% of particles for this compound are in the 50–250 nm range. Further increase in La^3+^ amount up to 25% resulted in a decrease in particle size (Figure 5b). For this sample, 95% of particles are in the 20–100 nm range. The Bi_0.5_La_0.5_Fe_0.85_Mn_0.15_O_3_ sample has analogous particle size distribution in comparison with a previous solid solution with 25% of La^3+^. With further increase in the concentration of La^3+^ ions, the particle size also increased, which can be seen in Figure 5d. For this Bi_0.1_La_0.9_Fe_0.85_Mn_0.15_O_3_ compound, nearly 92% of particles are in the 40–240 nm range. Interestingly, it was shown that an increase in Bi^3+^ content in Bi_x_La_1-x_MnO_3+δ_ solid solutions could lead to drastic particle growth [46]. On the other hand, our previous study revealed that Mn^3+^ could act as a particle growth inhibitor, limiting the grain size [22]. The intercalation of 15% Mn^3+^ ions limited the effect of particle growth, which could be related to the higher concentration of Bi^3+^ ions.

**Figure 6:**
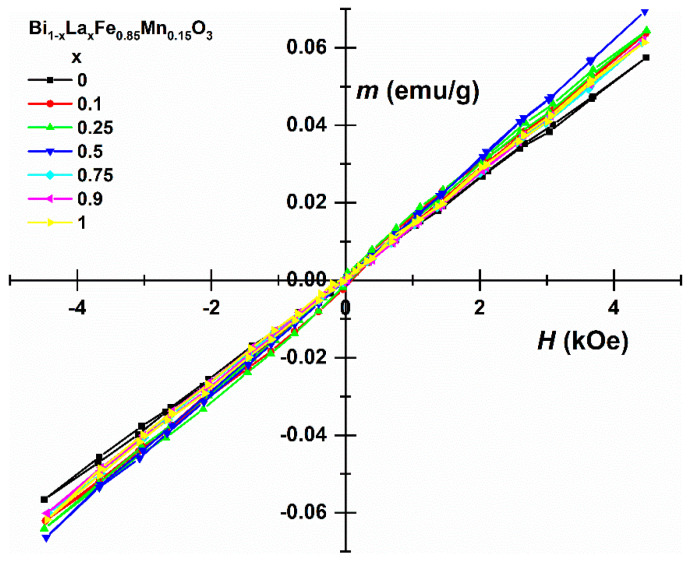
Hysteresis loops of Bi_1-x_La_x_Fe_0.85_Mn_0.15_O_3_ samples.

The linear dependence of magnetisation (Figure 6) is generally consistent with the antiferromagnetic order of spins in samples, but at least for BiFe_0.85_Mn_0.15_O_3_, the observed magnetisation is larger than that of bulk BiFeO_3_ [47,48]. It is noteworthy that in the case of nanocrystalline BiFeO_3_ and LaFeO_3_ (Table 1), weak ferromagnetism due to uncompensated magnetic moments on the surface of grains can cause an increase in magnetisation [49,50,51]. However, for BiFe_1-x_Mn_x_O_3_ solutions, Mn influences magnetic order, which can cause the formation of additional paramagnetic compounds [22], which cause a decrease in uncompensated magnetisation.

**Figure 7:**
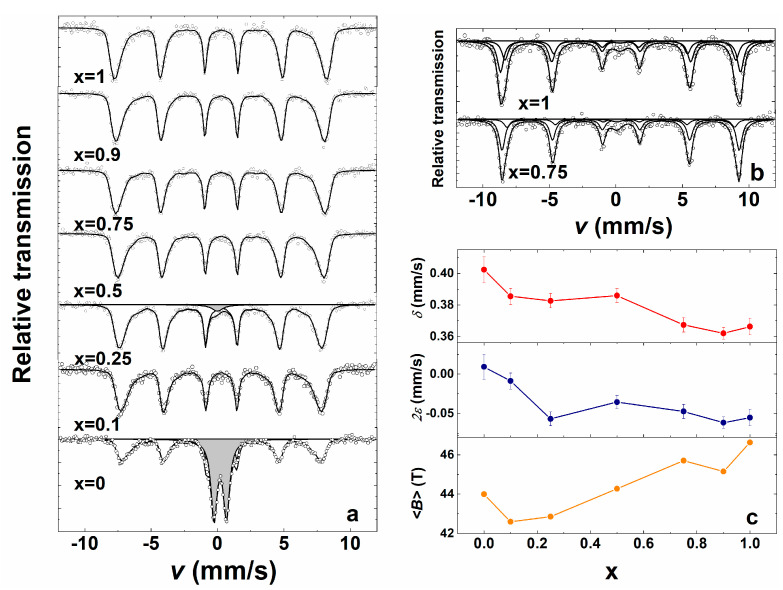
Mössbauer spectra of Bi_1-x_La_x_Fe_0.85_Mn_0.15_O_3_ samples at 296 K (**a**) and 10 K (**b**) and dependence of parameters: isomer shift δ and quadrupole shift 2ε, and average hyperfine field <B> of magnetically split part at 296 K on x (**c**).

Mössbauer spectra of Bi_1-x_La_x_Fe_0.85_Mn_0.15_O_3_ samples at room temperature (Figure 7a,b) showed broadened sextet lines, the increased width of which can be mostly related to the substitution of Fe by Mn. However, superparamagnetic relaxation of nanosized grains could also add to the line broadening here. Magnetically split part of spectra was fitted using hyperfine field distribution P(*B*) to account for line broadening. At low temperature (Figure 7b), however, the spectra could be fitted to two sextets. The line broadening caused the average hyperfine field <B> (Figure 7c) to be lower than the hyperfine field characteristic of BiFeO_3_ (49-50 T [48]) and LaFeO_3_ (51.3 T [51]). The parameters of Mössbauer spectra (Figure 7c) slightly depend on the composition of samples. A considerable amount of paramagnetic doublet (≈51% of the spectral area) appeared only for BiFe_0.85_Mn_0.15_O_3_. This doublet could be probably attributed to poor crystalline Bi_2_Fe_4_O_9_ as isomer shift δ = 0.31 ± 0.01 mm/s of the doublet is equal to the average isomer shift of two doublets characteristic of Bi_2_Fe_4_O_9_ [52].

## 4. Conclusions

An aqueous sol-gel method was successfully applied for the preparation of Bi_1-x_La_x_Fe_0.85_Mn_0.15_O_3_ (where 0 ≤ x ≤ 1) solid solution specimens. Nearly all compounds were phase-pure, with the exception of BiFe_0.85_Mn_0.15_O_3_, where the presence of Bi_2_Fe_4_O_9_ and Bi_25_FeO_40_ impurity phases was determined from X-ray diffraction analysis data and Mössbauer measurements. Depending on La^3+^ concentration, few structural transitions from orthorhombic to cubic and back to orthorhombic were observed in the series. Raman and FT-IR spectroscopies also provided additional structural information in good agreement with XRD analysis results. SEM analysis demonstrated that intercalation of Mn^3+^ ions limited the growth of the particles, and the largest particles were observed for Bi_0.9_La_0.1_Fe_0.85_Mn_0.15_O_3_. All samples demonstrated antiferromagnetic behaviour, with the highest magnetisation values observed for the Bi_0.5_La_0.5_Fe_0.85_Mn_0.15_O_3_ sample.

## Figures and Tables

**Table 1:** Crystallite size for Bi_1-x_La_x_Fe_0.85_Mn_0.15_O_3_ solid solutions.

Formula	D, nm
BiFe_0.85_Mn_0.15_O_3_	19.18
Bi_0.9_La_0.1_Fe_0.85_Mn_0.15_O_3_	20.39
Bi_0.75_La_0.25_Fe_0.85_Mn_0.15_O_3_	25.18
Bi_0.5_La_0.5_Fe_0.85_Mn_0.15_O_3_	28.72
Bi_0.25_La_0.75_Fe_0.85_Mn_0.15_O_3_	29.39
Bi_0.1_La_0.9_Fe_0.85_Mn_0.15_O_3_	19.60
LaFe_0.85_Mn_0.15_O_3_	29.53
